# Reliability and validity of the ESRD Symptom Checklist – Transplantation Module in Norwegian kidney transplant recipients

**DOI:** 10.1186/1471-2369-7-17

**Published:** 2006-11-16

**Authors:** Knut Stavem, Rüdiger Ganss

**Affiliations:** 1 Medical Department, Akershus University Hospital, Lørenskog, Norway; 2 Helse-Øst Health Services Research Centre, Lørenskog, Norway

## Abstract

**Background:**

The aim of the study was to validate the Norwegian version of a self-administered 43-item questionnaire designed to assess quality of life in kidney transplant recipients, the End-Stage Renal Disease Symptom Checklist – Transplantation Module (ESRD-SCL).

**Methods:**

In total, 53 kidney transplant recipients from one university-affiliated hospital responded to a questionnaire including the ESRD-SCL and the Short Form 36 (SF-36). We assessed internal consistency reliability and test-retest reliability with 2 weeks between assessments. Construct validity was assessed by correlations of the ESRD-SCL subscales with related and unrelated SF-36 scales, demographic, and clinical characteristics.

**Results:**

Subscales of the ESRD-SCL showed good internal consistency reliability (Cronbach's = 0.72–0.81) and for the aggregate total scale α was 0.94. Test-retest reliability median 14 days apart was excellent with intraclass coefficients ranging from 0.87 to 0.95. The pattern of correlations of the ESRD-SCL scales with related and unrelated scales SF-36 scales and demographic and clinical characteristics gave support to the construct validity of the ESRD-SCL.

**Conclusion:**

The Norwegian translation of the ESRD-SCL showed satisfactory internal consistency reliability, test-retest reliability and construct validity, at the level of the original German version.

## Background

Kidney transplantation has a positive impact on survival and morbidity of patients with end-stage renal disease. Recently, there has been increasing attention to health-related quality of life (HRQoL) as an important consideration in evaluating the impact of kidney transplantation. Kidney transplant recipients have better HRQoL than transplant candidates maintained on hemodialysis [1,2], and they experience an improvement of HRQoL in the first months after transplantation [3,4].

The HRQoL in kidney transplantation can be evaluated using generic tools, such as the Sickness Impact Profile (SIP), the Nottingham Health Profile (NHP) or the Short Form 36 (SF-36) [5]. Some disease-specific tools for evaluation of HRQoL in relationship to renal transplantation have been developed, including the Kidney Transplant Questionnaire [6], and the ESRD symptom checklist – transplantation module (ESRD-SCL) questionnaire [7]. Also, the Kidney Disease-Quality of Life (KDQOL) questionnaire is commonly used [8-10], although it was not developed specifically for the evaluation of transplantation.

To conduct studies on HRQoL outcomes in kidney transplant patients, it is necessary to adapt relevant questionnaires and tools to the appropriate language and assess the psychometric properties of the questionnaires in relevant populations. Based on content analysis and available documentation, we identified the ESRD-SCL as a feasible questionnaire for use in kidney transplantation, in particular to evaluate the effects of immunosuppressant medication [11].

The objective of the present study was to assess the reliability and construct validity of a Norwegian version of the ESRD-SCL in renal transplant recipients.

## Methods

### Subjects and study design

Kidney transplantation in Norway is centralized to Rikshospitalet University Hospital, Oslo; however, follow up is decentralized and performed in several hospitals. In the present study we aimed at including all consecutive kidney transplant recipients in a population of about 65 recipients, who regularly visits the nephrology outpatient clinic at Akershus University Hospital, irrespective of medication and time since transplantation. During an outpatient visit, the attending physician asked the patients to participate and to give their written informed consent. The participants received a package of self-administered questionnaires to be filled in at home and mailed to the study organizers. About 2 weeks after returning the questionnaires, the respondents received another identical questionnaire by mail. We contacted eight of the participants by telephone, as a reminder after the first questionnaire administration. No reminders were used for the retest.

We aimed at including 60 subjects in order to have data from 50 subjects for analysis. In total 59 patients accepted to participate; 53 returned the first questionnaire (90%), and 48 the second questionnaire (81%). The Regional committee for medical research ethics, Health Region East (REK Øst) approved the study, and the participants signed a declaration of informed consent.

### Questionnaires

The package of questionnaires contained the following instruments:

#### The ESRD symptom checklist – transplantation module (ESRD-SCL)

The ESRD-SCL was developed in Essen, Germany to assess quality of life after renal transplantation, focusing on transplantation-specific symptoms, side effects of immunosuppressive therapy and psychological distress [7]. It is available in German, English and Turkish versions [12]. The reliability, validity and responsiveness of this questionnaire have been assessed in a German population of renal transplantation patients [7,13], and the questionnaire has been used to compare HRQoL of patients using tacrolimus and ciclosporin-microemulsion [11].

The cultural and language adaptation of the ESRD-SCL was done according to a recommended procedure [14]. Two Norwegian physicians fluent in German in parallel translated the questionnaire into from German into Norwegian. A group with the two translators and two other members discussed the translations and agreed on a consensus version. This consensus version then was backtranslated into German by a physician fluent in Norwegian, but with German as his native language. The backtranslated version was compared with the original and discussed with the authors of the original version. The questionnaires were considered conceptually and linguistically equivalent. Before using the questionnaire, it was pilot-tested in some patients with kidney failure and found to be acceptable. The final Norwegian version of the questionnaire is presented as an appendix [see Additional file 1].

#### Short Form 36 (SF-36)

The general health status questionnaire SF-36 intends to assess aspects of health important to all patients. The SF-36 is developed in the U.S., contains eight scales [15,16], and two component summary scales, and has been extensively validated; however with limited experience in kidney transplant recipients. We used the Norwegian standard SF-36 version 1.2 [17], assessing health status during the past four weeks. The scales were scored from 0 (lowest level of functioning) to 100 (highest level of functioning). The SF-36 has previously been used in Norwegian kidney transplant recipients [18], and its psychometric properties have been demonstrated in Norwegian patients and scores for a normal population presented [17,19].

### Demographic and clinical variables

At study entry, the attending physician recorded information on the patients's renal disease, previous transplantations, time since transplantation, current medication, data from the most recent physical examination including height, weight, blood pressure, and comorbidity using Charlson's comorbidity index, and some laboratory tests (Hemoglobin, S-creatinine). In the self-administered questionnaire, the patients reported some supplementary demographic data, such as marital status, education, and employment status.

### Statistical analysis

Descriptive statistics are presented using the mean (SD), median (range) or numbers (%). Internal consistency reliability was assessed using Cronbach's α [20]. Test-retest reliability was assessed over 2 weeks with an intraclass correlation coefficient (ICC), using the average of raters in a two-way mixed model ICC with absolute agreement definition.

Construct validity for the ESRD-SCL subscales was assessed by comparing actual correlations with the SF-36 scales with apriori predicted correlations. Based on previous literature [7,21] and content analysis of the items of the scales, we hypothesized that:

(1) The two ESRD-SCL dimensions Limited cognitive capacity and Transplantation-associated psychological distress would have the highest correlations with the SF-36 scale Mental health.

(2) The correlation of the ESRD-SCL dimension Cardiac and renal dysfunction would be highest with the SF-36 scale Physical functioning.

(3) The correlations between the two scales measuring medication side effects and the SF-36 scales would be weak.

(4) Associations with sociodemographic and clinical data would be highest for employment, age, sex, time since transplantation, and comorbidity, possibly in the range 0.08–0.30 roughly equivalent to an incremental R^2 ^of 0.006–0.09 [7]. For comorbidity we used the Charlson comorbidity index (range 2–8) [22]. We also investigated the association with Hemoglobin, S-creatinine and immunosuppressive regimen (tacrolimus/prednisolone vs. ciclosporin/prednisolone), possibly being able to capture the characteristic side effects of ciclosporin of gum and hair growth.

For correlations between the ESRD-SCL and SF-36 scales we used Pearson's correlation coefficient. For correlations of the ESRD-SCL scales with demographic and clinical variable we used Spearman's rank correlations, because of the ordinal nature of some of the data and the dichotomy of some variables.

Finally, we assessed the capacity of the questionnaire subscales subscales to discriminate between two known groups, as defined by: (1) immunosuppressive regimen, comparing tacrolimus/prednisolone (n = 9) with ciclosporin/prednisolone (n = 39); (2) Age below/above the sample median of 57.87 years; (3) comorbidity, using Charlson comorbidity index above/below the median (≤2 vs >2). These or similar variables have been associated with HRQoL following renal transplantation in previous studies [7,21]. In this analysis we used analysis of variance/covariance, adjusting for age, sex and comorbidity (Charlson comorbidity index ≤2 vs >2), where appropriate.

Sample size for test-retest analysis was planned to about 50 patients, which is commonly used in the literature. However, formal sample size calculation for test-retest analysis or correlation analysis is rarely done and was not carried out in the present study. We chose a 5% significance level, using two-sided tests. The SPSS statistical software version 12.0 (SPSS Inc., Chicago, IL) was used for all analyses.

## Results

The characteristics of the 59 respondents are shown in Table 1. ESRD-SCL and SF-36 scores are shown in Table 2, including the percentage of respondents giving lowest (floor) and highest possible score (ceiling). The ESRD-SCL scores did not concentrate at the ceiling for any subscale. However, scores concentrated at the floor for the Limited cognitive capacity, Side effects of corticosteroids, and Increased growth of gum and hair subscales (Table 2). For the SF-36 scales role – physical 30% of respondents scored the lowest possible value (floor). There were marked ceiling effects on the SF-36 scales role – physical (40%), bodily pain (25%), social functioning (50%), and role – emotional (67%) scales (Table 2).

**Table 1:** Respondents' characteristics (n = 53), mean (SD) unless otherwise stated

Age, years	57.5 (13.2)
Female gender, number (%)	22 (42)
Married/cohabiting, number (%)	40 (76)
Education in years, number (%)	
<10	17 (32)
10–12	22 (42)
>12	12 (26)
Body mass index, kg/m^2^	26.8 (5.8)
Systolic blood pressure, mmHg	131 (15)
Diastolic blood pressure, mmHg	78 (8)
S-creatinine, μmol/L	136 (74)
Hemoglobin, g/dL	13.6 (1.6)
Employment, number (%)	
Full-time employed	18 (34)
Part-time employed	3 (6)
On sick-leave	4 (8)
Disability pension	15 (28)
Retired because of age	11 (21)
Other	
Diagnosis, number (%)	
Glomerulonephritis	19 (36)
Polycystic disease	14 (26)
Hypertensive renal disease	7 (13)
Diabetic nephropathy	7 (13)
Other	6 (12)
Type of transplantation, number (%)	
Cadaveric	32 (60)
Living donor	21 (40)
Immunosuppression, number (%)	
Ciclosporin A	40 (76)
Tacrolimus	10 (19)
Mycophenolate mofetil	20 (38)
Azathioprine	14 (26)
Prednisolone	51 (96)
Blood pressure medications, number (%)	
0	18 (34)
1	9 (17)
2	14 (26)
3+	12 (23)
Charlson comorbidity index, median (range)	2 (2–8)
Time since transplantation, years (median (range))	4.4 (0.4–29.1)

**Table 2:** Health-related quality of life scores and relaibility assessments

		Scores							Reliability	
			
	n	Items	Minimum	Maximum	Mean	SD	%min (floor)	%max (ceiling)	Internal consistency*	Test-retest**
**ESRD-SCL (range 0–4)**										
Limited physical capacity	53	10	0.10	2.50	0.74	0.54	0	0	0.80	0.95
Limited cognitive capacity	53	8	0	2.00	0.56	0.48	13	0	0.80	0.93
Cardiac and renal dysfunction	53	7	0	2.57	0.71	0.59	6	0	0.81	0.90
Side effects of corticosteroids	53	5	0	2.20	0.60	0.57	21	0	0.72	0.94
Increased growth of gum and hair	53	5	0	3.80	0.72	0.80	13	0	0.80	0.87
Transplantation-associated psychological distress	53	8	0	1.88	0.59	0.46	6	0	0.78	0.87
Total	53	43	0.07	2.07	0.65	0.43	0	0	0.94	0.95
**SF-36 (range 0–100)**										
Physical functioning	53	10	20	100	73.1	24.5	0	13	0.91	0.87
Role – physical	53	5	0	100	54.7	43.3	30	40	0.89	0.88
Bodily pain	52	2	0	100	65.3	28.7	2	25	0.91	0.93
General health	51	5	0	100	57.5	26.0	2	2	0.85	0.95
Vitality	52	4	5	95	54.4	21.0	0	0	0.80	0.90
Social functioning	52	2	12.5	100	81	24.6	0	50	0.84	0.86
Role – emotional	51	4	0	100	78.4	34.5	0	67	0.80	0.83
Mental health	52	5	24	100	77.8	17.9	0	10	0.81	0.91

* Cronbach's α

** Intraclass correlation coefficient

The Cronbach's α was high for all dimensions of the ESRD-SCL (α = 0.72–0.81), for the total scale (α = 0.94) (Table 2), and for the scales of the SF-36 (α = 0.80–0.91). In the test-retest the respondents (n = 48) completed questionnaires median 14 days apart (interquartile range 9 to 20 days). In the test-retest, the intraclass correlation coefficients for the different subscales of the ESRD-SCL ranged 0.87 to 0.95, and for the SF-36 from 0.83 to 0.95.

In the assessment of construct validity, the hypothesized associations between scales of the ESRDL-SCL generally were among the highest, largely confirming the hypothesis, although some of the other subscales also correlated well (Table 3). The correlations of the two scales measuring medication side effects with the SF-36 scales were among the weakest pairwise correlations.

**Table 3:** Multitrait-multimethod correlation matrix. Pearson's correlation coefficients between subscales of the ESRD-SCL and SF-36

	**ESRD-SCL**							
	
	n	Limited physical capacity	Limited cognitive capacity	Cardiac and renal dysfunction	Side effects of corticosteroids	Increased growth of gum and hair	Transplantation-associated psychological distress	Total
**SF-36**								
Physical functioning	53	-0.54**	-0.38**	-0.41**	-0.38**	-0.26	-0.38**	-0.52**
Role – physical	53	-0.52**	-0.43**	-0.28*	-0.04	-0.14	-0.27	-0.39**
Bodily pain	52	-0.74**	-0.51**	-0.52**	-0.33*	-0.31*	-0.50**	-0.65**
General health	51	-0.59**	-0.41**	-0.55**	-0.32*	-0.29*	-0.38**	-0.57**
Vitality	52	-0.69**	-0.49**	-0.44**	-0.26	-0.39**	-0.36**	-0.60**
Social functioning	52	-0.58**	-0.41**	-0.28*	-0.24	-0.16	-0.54**	-0.50**
Role – emotional	51	-0.67**	-0.44**	-0.31*	-0.24	-0.25	-0.52**	-0.55**
Mental health	52	-0.54**	-0.51**	-0.15	-0.15	-0.02	-0.65**	-0.46**

*<0.05 **<0.01

Among the demographic and clinical variables, employment showed the highest correlation with most of the ESRDL-SCL subscales, although these correlations were all weak and <0.40 (Table 4). Increased growth of gum and hair, a typical side effect of ciclosporin, was moderately associated with a ciclosporin-containing immunosuppressive regimen. With the above exceptions, associations of the ESRDL-SCL subscales with demographic and clinical variables were weak or close to nothing, as hypothesized (Table 4).

**Table 4:** Spearman's rank correlation coefficients of subscales of the ESRD-SCL with demographic and clinical variables

	n	Limited physical capacity	Limited cognitive capacity	Cardiac and renal dysfunction	Side effects of corticosteroids	Increased growth of gum and hair	Transplantation-associated psychological distress	Total
Age (years)	53	0.22	0.18	0.18	-0.13	0.08	-0.06	0.09
Female (2) vs. male (1)	53	0.10	-0.04	0.25	0.34*	-0.05	0.07	0.17
Employment (yes/no)	53	-0.38**	-0.30*	-0.23	-0.19	-0.37**	-0.15	-0.34*
Time since transplantation (years)	53	0.19	-0.01	0.14	-0.17	0.14	-0.21	0.02
Charlson's comorbidity index	53	0.24	0.19	0.20	0.04	0.03	0.06	0.17
Hemoglobin (g/dL)	53	-0.14	-0.01	-0.29*	-0.13	0.11	-0.18	-0.20
S-creatinine (umol/L)	53	0.16	0.11	0.20	-0.04	-0.06	0.13	0.15
CsA (2) vs. Tacrolimus (1)	48	0.08	0.06	0.06	-0.05	0.48**	-0.11	0.08
No. of drugs for hypertension	53	0.15	0.08	0.15	0.03	-0.22	0.17	0.14

*<0.05 **<0.01

Only the ESRD-SCL subscale Increased growth of gum and hair discriminated between patients with two different immunosuppressive regimens after adjustment for age, sex, and comorbidity (Table 5). Only the two SF-36 scales Role – physical and Bodily pain discriminated between patients below/above the median age in the multivariate model. On the ESRD-SCL subscale Side effects of corticosteroids, younger patients tended indicate more problems than those above the median age, although this difference was statistically not significant (Table 5). The SF-36 physical functioning scale was the only scale that discriminated between patient groups according to Charlson comorbidity index ≤2 vs. >2 (p =< 0.001). The ESRD-SCL subscales Limited physical capacity and Cardiac and renal dysfunction showed differences that were almost statistically significant (Table 5), however, we hypothesized that these scales were associated with the Physical functioning scale of the SF-36.

**Table 5:** Health-related quality of life scores according to immunosuppressive regimen, age below/above the median and comorbidity below/above the median

	Ciclosporin*			Age (years)**			Charlson comorbidity index***		
			
	Tacrolimus and prednisolon	Ciclosporin and prednisolon	p	≤58.88	>58.88	p	≤2	>2	p
	
N	9	39		27	26		30	23	
**ESRD-SCL**									
Limited physical capacity	0.62 ± 0.18	0.82 ± 0.09	0.33	0.73 ± 0.11	0.81 ± 0.11	0.62	0.63 ± 0.10	0.91 ± 0.11	0.06
Limited cognitive capacity	0.45 ± 0.15	0.59 ± 0.08	0.43	0.57 ± 0.10	0.58 ± 0.10	0.93	0.46 ± 0.09	0.69 ± 0.10	0.10
Cardiac and renal dysfunction	0.72 ± 0.19	0.82 ± 0.08	0.21	0.73 ± 0.11	0.79 ± 0.11	0.71	0.62 ± 0.10	0.91 ± 0.12	0.07
Side effects of corticosteroids	0.67 ± 0.18	0.68 ± 0.09	0.20	0.71 ± 0.11	0.58 ± 0.11	0.16	0.53 ± 0.10	0.76 ± 0.12	0.16
Increased growth of gum and hair	0.19 ± 0.27	0.92 ± 0.09	0.02	0.74 ± 0.16	0.73 ± 0.16	0.98	0.61 ± 0.15	0.87 ± 0.17	0.26
Transplantation-associated psychological distress	0.67 ± 0.16	0.58 ± 0.08	0.60	0.66 ± 0.09	0.54 ± 0.09	0.38	0.56 ± 0.09	0.64 ± 0.10	0.56
Total	0.57 ± 0.14	0.73 ± 0.07	0.32	0.69 ± 0.08	0.68 ± 0.09	0.95	0.57 ± 0.08	0.80 ± 0.09	0.07
**SF-36**									
Physical functioning	71.3 ± 6.5	73.8 ± 3.2	0.74	73.5 ± 4.3	68.1 ± 4.3	0.38	81.7 ± 3.8	59.9 ± 4.3	<0.001
Role – physical	70.6 ± 13.4	50.4 ± 6.6	0.18	70.3 ± 8.2	38.0 ± 8.3	0.009	55.6 ± 7.2	52.7 ± 8.2	0.80
Bodily pain	58.8 ± 9.4	65.2 ± 4.7	0.54	72.2 ± 5.6	55.3 ± 5.6	0.04	66.9 ± 5.0	61.0 ± 5.8	0.44
General health	49.6 ± 8.8	56.0 ± 4.5	0.52	61.0 ± 5.2	51.6 ± 5.2	0.21	60.8 ± 4.9	51.8 ± 5.5	0.23
Vitality	61.6 ± 7.3	51.0 ± 3.7	0.20	58.4 ± 4.2	48.7 ± 4.2	0.11	57.4 ± 3.9	49.8 ± 4.6	0.22
Social functioning	70.3 ± 8.1	81.3 ± 4.1	0.23	81.7 ± 4.9	77.9 ± 5.0	0.6	85.4 ± 4.5	74.3 ± 5.2	0.12
Role – emotional	71.1 ± 11.4	76.8 ± 5.7	0.66	85.4 ± 6.7	67.3 ± 7.1	0.08	81.4 ± 6.4	72.0 ± 7.3	0.35
Mental health	73.3 ± 6.2	76.7 ± 3.1	0.62	78.1 ± 3.6	76.4 ± 3.7	0.75	79.8 ± 3.4	74.7 ± 3.9	0.34

*adjusted for age, sex and comorbidity

** adjusted for sex and comorbidity

*** adjusted for age and sex

## Discussion

In this cross-sectional study, the Norwegian version of the ESRD-SCL demonstrated high internal consistency reliability for all six subscales, in line with the original German version (α = 0.76–0.85) [7]. Furthermore the test-retest reliability over 2 weeks for all scales was excellent. Reproducibility for the scales of this questionnaire has previously only been assessed over 1 year in stable patients [7]. It has been suggested that HRQoL instruments can be used for comparisons at group level if reliability is above 0.70 [23]. All subscales of both the ESRD-SCL and the SF-36 instruments had higher internal consistency reliability than this. For use at the level of the individual patient, a suggested minimum requirement for reliability is 0.90 while 0.95 is desirable [23] although perhaps too stringent [24].

The pattern of correlations between the two instruments largely confirmed hypothesized associations from literature review and item content analysis, hence supporting convergent and discriminant validity of the ESRD-SCL [25]. Furthermore, the associations of the ESRD-SCL subscales with demographic and clinical variables were in line with expectations. The patients accepted the questionnaire well, as demonstrated by the high completion rates.

We found no difference in HRQoL between a tacrolimus-based and a ciclosporin-based regimen using the SF-36, and no systematic difference on the ESRD-SCL subscales except the Increased growth of gum an hear subscale, however our sample was small. Previous studies have reported comparable effects on global HRQoL of the two regimens, while Tacrolimus has tended to improved the disease-specific HRQoL [11,26-30].

A previous study has reported lower scores, denoting less side effects, on the Side effects of corticosteroids dimension of the ESRD-SCL and more suffering in the Limited cognitive function and Increased growth of gum and hair dimension among the elderly [7]. In the present study we noted a statistically nonsignificant tendency to lower Side effects of corticosteroids in the elderly, however there was no indication of a difference according to age in the Limited cognitive dimension or Increased growth of gum and hair dimension in the present study.

Some weaknesses of the study should be noted. This study was a cross-sectional study in one hospital with a limited sample size, and all patients were successful renal transplant recipients.

The questionnaire contains items intended to assess side effects of immunosuppressive medication. Hence, the questionnaire can be a useful supplement to other questionnaires in studies of kidney transplantation, in particular in studies of variations in immunosuppressive medication following transplantation.

## Conclusion

In summary, we have demonstrated that the psychometric properties of the Norwegian version of the ESRD-SCL were satisfactory and in line with the German original. Hence, the questionnaire can be recommended for use in future studies. The present study was not designed to evaluate responsiveness, which should be assessed in a longitudinal study.

## Competing interests

The author(s) declare that they have no competing interests.

## Authors' contributions

KS participated in planning and design of the study, organization of data collection, performed the statistical analysis, drafted and revised the manuscript. RG participated in the planning and design of the study, organization of data collection, and critically reviewed the manuscript. Both authors read and approved the final manuscript.

## Pre-publication history

The pre-publication history for this paper can be accessed here:



## Supplementary Material

Additional file 1 The Norwegian version of the End-Stage Renal Disease Symptom Checklist-Transplantation Module (ESRD-SCL). The additional file shows the Norwegian version of the ESRD-SCL, which was translated and validated in the article. Click here for file
