# Supplementary material for: Reliability and validity of the ESRD Symptom Checklist – Transplantation Module in Norwegian kidney transplant recipients
Source: BMC Nephrol. 2006 Nov 16;7:17. doi: 10.1186/1471-2369-7-17 (PMC1660533; doi:10.1186/1471-2369-7-17)
Supplement: Additional file 1 — The Norwegian version of the End-Stage Renal Disease Symptom Checklist-Transplantation Module (ESRD-SCL). The additional file shows the Norwegian version of the ESRD-SCL, which was translated and validated in the article. [file 1471-2369-7-17-S1.doc]

### Additional file 1

**Appendix:** The Norwegian version of the End-Stage Renal Disease Symptom Checklist-Transplantation Module (ESRD-SCL).

ESRD-SCL^TM^. Listen nedenfor presenterer mange plager som av og til kan ramme kropp og sjel. Vennligst kryss av for det tallet ved hvert utsagn som passer best for deg. Vennligst svar på alle spørsmål!

I hvilken grad plages du for tiden av….

| Kategori | Overhodet  ikke | Litt | Ganske  mye | Sterkt | Svært  sterkt |
| --- | --- | --- | --- | --- | --- |
| Tall | 0 | 1 | 2 | 3 | 4 |

| 01. | Dårlig helsetilstand | 0-1-2-3-4 |
| --- | --- | --- |
| 02. | Nedsatt fysisk yteevne | 0-1-2-3-4 |
| 03. | Utseendet ditt | 0-1-2-3-4 |
| 04. | Nedsatt mental yteevne | 0-1-2-3-4 |
| 05. | Tanker omkring transplantasjon/din transplantasjon | 0-1-2-3-4 |
| 06. | Grubling omkring organgiveren | 0-1-2-3-4 |
| 07. | Spørsmålet om hvor lenge transplantatet vil fungere | 0-1-2-3-4 |
| 08. | Mareritt | 0-1-2-3-4 |
| 09. | Hodepine | 0-1-2-3-4 |
| 10. | Søvnløshet | 0-1-2-3-4 |
| 11. | Dårlig humør | 0-1-2-3-4 |
| 12. | Konsentrasjonsforstyrrelser | 0-1-2-3-4 |
| 13. | Nervøsitet | 0-1-2-3-4 |
| 14. | Kraftig svimmelhet | 0-1-2-3-4 |
| 15. | Angsttilstander | 0-1-2-3-4 |
| 16. | Glemsomhet | 0-1-2-3-4 |
| 17. | Dårligere syn | 0-1-2-3-4 |
| 18. | Dårligere hørsel | 0-1-2-3-4 |
| 19. | Øresus | 0-1-2-3-4 |
| 20. | Humørsvingninger | 0-1-2-3-4 |
| 21. | Hjerterytmeforstyrrelser | 0-1-2-3-4 |
| 22. | For høyt blodtrykk | 0-1-2-3-4 |
| 23. | Skjelettsmerter | 0-1-2-3-4 |
| 24. | Leddsmerter | 0-1-2-3-4 |
| 25. | Muskelsmerter | 0-1-2-3-4 |
| 26. | Forkjølelse eller influensa | 0-1-2-3-4 |
| 27. | Økt kroppsbehåring | 0-1-2-3-4 |
| 28. | Vekst av tannkjøttet | 0-1-2-3-4 |
| 29. | Tannkjøttblødninger | 0-1-2-3-4 |
| 30. | Oppsvulmet ansikt | 0-1-2-3-4 |
| 31. | Økte infeksjoner | 0-1-2-3-4 |
| 32. | Hovne føtter | 0-1-2-3-4 |
| 33. | Magesmerter | 0-1-2-3-4 |
| 34. | Endret følelse i bena | 0-1-2-3-4 |
| 35. | Følelsen av å være utslått eller utmattet | 0-1-2-3-4 |
| 36. | Tannkjøttforandringer | 0-1-2-3-4 |
| 37. | Økt hårvekst | 0-1-2-3-4 |
| 38. | Hovent ansikt om morgenen | 0-1-2-3-4 |
| 39. | Vektforandringer | 0-1-2-3-4 |
| 40. | Hovne ben | 0-1-2-3-4 |
| 41. | Tendens til blåmerker | 0-1-2-3-4 |
| 42. | Økt tørste | 0-1-2-3-4 |
| 43. | Hukommelsesforstyrrelser | 0-1-2-3-4 |
